# OA11.01. Integrative care for the management of low back pain: design of a clinical care pathway

**DOI:** 10.1186/1472-6882-12-S1-O41

**Published:** 2012-06-12

**Authors:** M Maiers, K Westrom, C Legendre, G Bronfort

**Affiliations:** 1 Northwestern Health Sciences University, Bloomington, USA

## Purpose

For the treatment of chronic back pain, it has been theorized that integrative care plans can lead to better outcomes than those achieved by monodisciplinary care alone, especially when using a collaborative, interdisciplinary, and non-hierarchical team approach. This paper describes the development and implementation of a care pathway designed to guide treatment by an integrative group of providers within a randomized controlled trial.

## Methods

A clinical care pathway was used by a multidisciplinary group of providers, which included acupuncturists, chiropractors, cognitive behavioral therapists, exercise therapists, and primary care physicians. Treatment recommendations were based on an evidence-informed practice model, and reached by group consensus. Research study participants were empowered to select one of the treatment recommendations proposed by the integrative group. Common principles and benchmarks were established to guide treatment management throughout the study.

## Results

Thirteen providers representing 5 healthcare professions collaborated to provide integrative care to study participants. On average, 3 to 4 treatment plans, each consisting of 2 to 3 modalities, were recommended to study participants. Exercise, massage, and acupuncture were both most commonly recommended by the team and selected by study participants. Over one-third of treatment plans were re-evaluated over the course of care by the integrative team; changes most commonly incorporated cognitive behavioral therapy to care.

## Conclusion

The integrative care pathway designed for this trial proved to be an essential mechanism to operationalize care, allowing team members and patients to consistently and effectively apply treatment plans. The pragmatic design of this research study required a high level of communication and flexibility between participants, providers, case managers, and project managers. Clinical care pathways can be useful tools in providing evidence-based treatment, especially in the context of multidisciplinary or integrative care settings.

